# Products of the visual cycle are detected in mice lacking retinol binding protein 4, the only known vitamin A carrier in plasma

**DOI:** 10.1016/j.jbc.2022.102722

**Published:** 2022-11-19

**Authors:** Diego Montenegro, Jin Zhao, Hye Jin Kim, Igor O. Shmarakov, William S. Blaner, Janet R. Sparrow

**Affiliations:** 1 Departments of Ophthalmology, Columbia University Medical Center, New York, New York, USA; 2 Departments of Medicine, Columbia University Medical Center, New York, New York, USA; 3 Departments of Pathology and Cell Biology, Columbia University Medical Center, New York, New York, USA

**Keywords:** retinol binding protein 4, bisretinoid lipofuscin, quantitative fundus autofluorescence, retina, retinal pigment epithelium, retinoids, ERG, electroretinogram, NIR-AF, near infrared autofluorescence, ONH, optic nerve head, ONL, outer nuclear layer, PE, phosphatidylethanolamine, qAF, quantitative fundus autofluorescence, RBP4, retinol binding protein 4, RPE, retinal pigment epithelium, SW-AF, short-wavelength fundus autofluorescence, TTR, transthyretin

## Abstract

Efficient delivery of vitamin A to the retinal pigment epithelium is vital to the production of the light-sensitive visual chromophore 11-*cis*-retinal. Nevertheless, retinol binding protein 4 (RBP4) is the only known carrier of vitamin A in plasma. Here, we present new findings that further characterize the visual cycle in the presence of *Rbp4* deficiency. In the face of impaired delivery of retinol in *Rbp4*^−/−^ mice, we determined that 11-*cis*-retinaldehyde reached levels that were ∼60% of WT at 4 months of age and all-*trans*-retinyl ester was 18% of normal yet photoreceptor cell loss was apparent by 8 months of age. The lack of Rbp4 appeared to have a greater impact on scotopic rod–mediated responses than on cone function at early ages. Also, despite severely impaired delivery of retinol, bisretinoid lipofuscin that forms as a byproduct of the visual cycle was measurable by HPLC and by quantitative fundus autofluorescence. In mice carrying an Rpe65 amino acid variant that slows visual cycle kinetics, Rbp4 deficiency had a less pronounced effect on 11-*cis*-retinal levels. Finally, we found that ocular retinoids were not altered in mice expressing elevated adipose-derived total Rbp4 protein (*hRBP4*^*+/+*^*AdiCre*^*+/−*^). In conclusion, our findings are consistent with a model in which vitamin A can be delivered to the retina by Rbp4-independent pathways.

The 11-*cis* form of vitamin A (all-*trans*-retinol) is the light sensitive chromophore of rods and cones. The 11-*cis*-retinaldehyde chromophore is generated within a multi-step pathway located in retinal pigment epithelium (RPE) and photoreceptor cells. Vitamin A is acquired from the diet as all-*trans*-retinol (retinol) or all-*trans*-retinyl ester (retinyl ester) and is absorbed and stored primarily in the liver as retinyl ester (1). Retinol-binding protein 4 (RBP4) is responsible for transporting retinol in plasma and to this end is secreted by liver and fat cells. Lesser amounts of RBP4 may be synthesized by RPE (2, 3, 4) but whether the protein reaches the systemic circulation is not known. In the circulation, RBP4 is normally bound to transthyretin (TTR); this complex transports serum retinol in a 1:1 ratio (5). It is estimated that 95% of the retinoid present in the nonprandial circulation is bound to RBP4/TTR (6). Some cells, such as RPE, express the cell surface receptor Stra6 (7, 8) that binds RBP4 for uptake of retinol into the cells. RPE cells are one of the cell types that accumulate and store relatively high levels of vitamin A in the form of retinyl esters (1). However, even in the absence of Rbp4 (*Rbp4*^−/−^), mice are reported to acquire levels of retinol sufficient to record a normal electroretinogram (ERG) at age 6 months (9) with a further increase between 3 and 10 months of age (10).

As a by-product of the visual cycle, deleterious vitamin A-aldehyde adducts (bisretinoids) form randomly and nonenzymatically by reaction of retinaldehyde with photoreceptor outer segment lipid—specifically, phosphatidylethanolamine (PE) (11). Bisretinoids are transferred to RPE cells within phagocytosed outer segment disks and accumulate as lipofuscin (12, 13, 14, 15, 16). Abundant evidence indicates that the rate of bisretinoid formation can be modulated by visual cycle kinetics. For instance, limiting delivery of retinol to RPE (17, 18) and reducing the activity of the isomerase Rpe65 by a gene variant (Rpe65-Leucine450Methionine) (19) or compound (20, 21, 22) reduces bisretinoid formation and protects against the adverse consequences of their accumulation. Since bisretinoids underlie the short-wavelength fundus autofluorescence (SW-AF, 488 nm excitation) that is measured *in vivo*, noninvasive quantitation of this emission serves to assess bisretinoid levels (23).

We have undertaken to study retinoid levels, retinal function, and bisretinoid accumulation in *Rbp4*^*−/−*^ mice. Taken together, these measures provide some indication of the retinol levels available to retina. We have also taken note of developmental anomalies in *Rbp4*^*−/−*^ mice and we examined an adipocyte tissue–specific human RBP4 knock-in mouse.

## Results

### Quantitation of ocular retinoids in *Rbp4*^*−/−*^ mice

**Figure 1:**
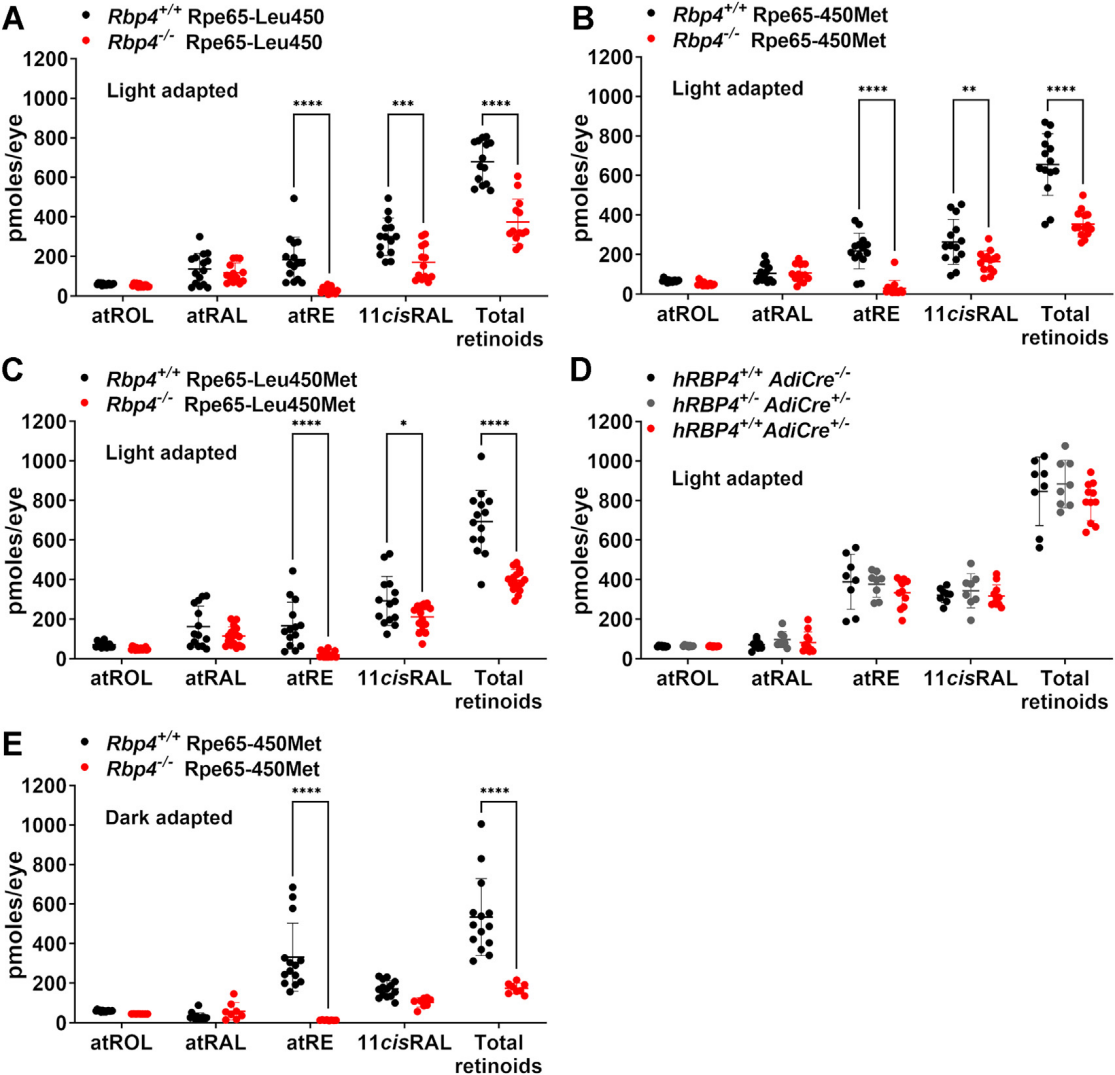
**UPLC quantitation of ocular retinoids in *Rbp4* knock-out and tissue specific *Rbp4* knock-in mice.** All-*trans*-retinol (atROL), all-*trans*-retinal (atRAL), all-*trans*-retinyl ester (atRE), 11-*cis*-retinal (11*cis*RAL). *A*–*C*, light-adapted mice were 4 months of age, had agouti coat color, and the Rpe65-Leucine450Methionine (Rpe65Leu450Met) variant was either Rpe65-Leu450 (*A*), Rpe65-450Met (*B*), or heterozygous (Rpe65-Leu450Met) (*C*) as indicated compared to WT *Rbp4*^*+/+*^ litter mates. *D*, mice expressing human RBP4 in adipose tissue (*hRBP4*^*+/+*^*AdiCre*^*+/−*^). Controls (*hRBP4*^*+/+*^*AdiCre*^*−/−*^, *hRBP4*^*+/−*^*AdiCre*^*+/−*^) are also presented. Mice were 4 months of age, had agouti coat color, were light-adapted, and were fed a high fat diet for 2 months. *E*, dark-adapted *Rbp4*^*−/−*^ compared to WT C57BL/6J-Aw-J/J mice at age 5 months, black coat color, and Rpe65-450Met variant. Individual values and mean ± SD are plotted. Each value is based on one eye. ∗ *p* <0.05, ∗∗ *p* <0.01, ∗∗∗ *p* <0.001, ∗∗∗∗ *p* <0.0001, 2-way ANOVA and Sidak’s multiple comparison test. RBP4, retinol binding protein 4.

The 11-*cis* isomer of vitamin A aldehyde is the chromophore of visual pigment capable of absorbing photons of light. Vitamin A is supplied to the visual cycle by RPE uptake from serum. Rbp4 is the only known transporter of vitamin A (retinol) in blood (5). At 4 months of age, both 11-*cis*-retinaldehyde and all-*trans*-retinyl ester were reduced in the *Rbp4*^*−/−*^ *versus Rbp4*^*+/+*^ mice (agouti, Rpe65Leu, standard diet, light-adapted); the 11-*cis* isomer was reduced by 43% (*p* < 0.01, 2-way ANOVA and Sidak’s multiple comparison test) while the ester was 82% lower than in *Rbp4*^*+/+*^ mice (*p* < 0.001, 2-way ANOVA and Sidak’s multiple comparison test) (Fig. 1*A*).

When the visual cycle isomerase Rpe65 carries methionine at amino acid 450 instead of leucine, regeneration of 11-*cis*-retinal is reduced perhaps because of Rpe65 protein stability (24, 25, 26). We found that in *Rbp4*^*−/−*^ mice that were homozygous for the Rpe65-450Met variant, the difference in 11-*cis*-retinal levels (*Rbp4*^*−/−*^ vs *Rbp4*^*+/+*^) was somewhat less pronounced (Fig. 1*B*). Specifically, the 11-*cis* isomer was reduced by 38% and the ester was 86% lower than in *Rbp4*^*+/+*^ mice (*p* < 0.01, 2-way ANOVA and Sidak’s multiple comparison test). Even when the mice were dark-adapted for 18 h to favor 11-*cis*-retinal regeneration, levels of the latter retinoid remained 40% lower in the Rbp4-deficient mice (Fig. 1*E*).

In a second analysis of mice at 4 months of age (agouti, light-adapted, Rpe65-Leu450Met heterozygous, 15 IU/gm vitamin A), 11-*cis* retinaldehyde was decreased by 38% as well (*p* < 0.01, 2-way ANOVA and Sidak’s multiple comparison test) in the *Rbp4*^*−/−*^ mice and all-*trans-*retinyl ester was reduced by 88% (*p* < 0.001, 2-way ANOVA and Sidak’s multiple comparison test) (Fig. 1*C*).

**Figure 2:**
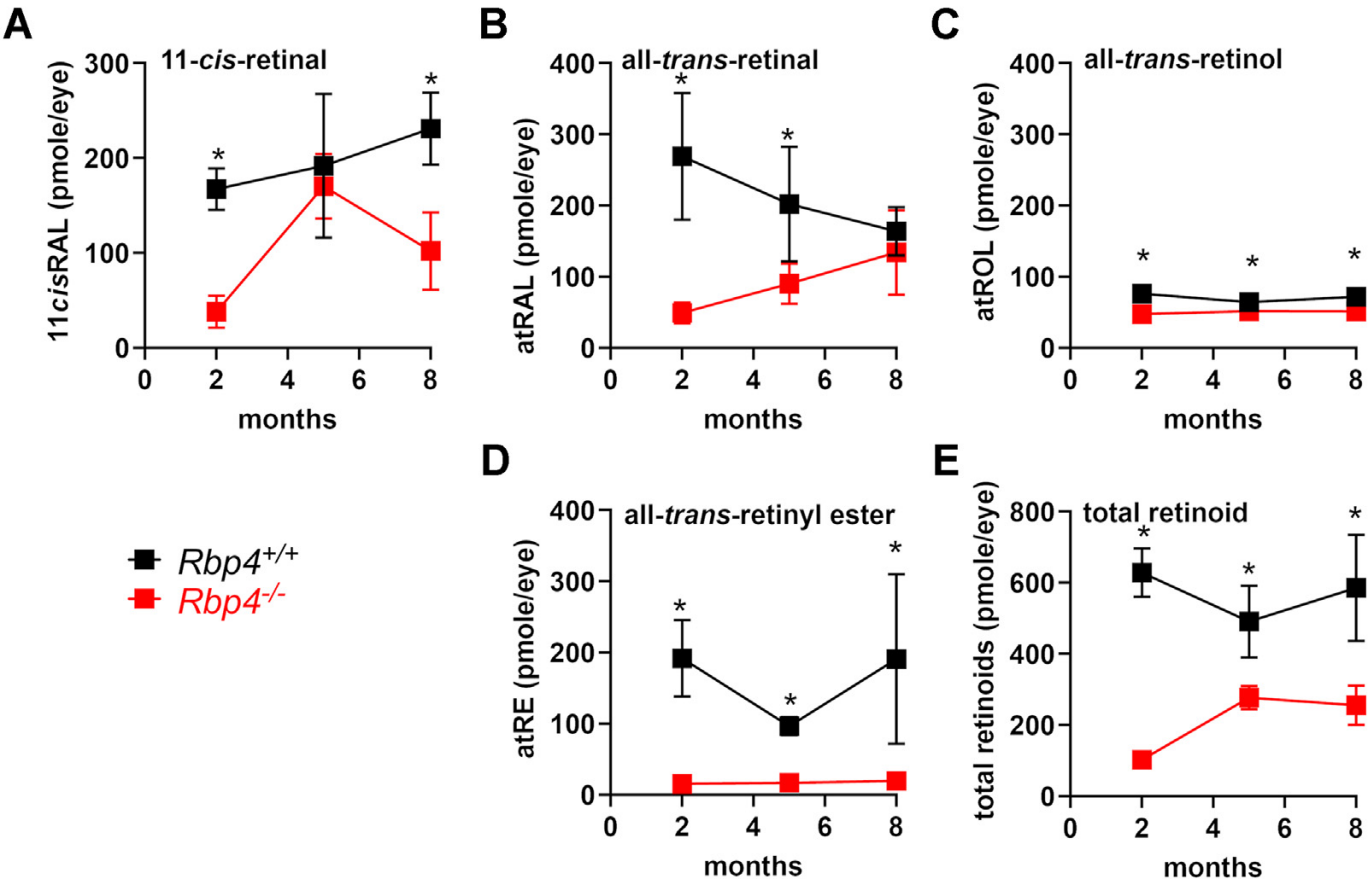
**Serial measurements of ocular retinoid at ages 2, 5, and 8 months in light-adapted agouti *Rbp4***^***−/−***^**(*red symbols*) and WT C57BL/6J-Aw-J/J mice (*black symbols*).** 11-*cis*-retinal (*A*), all-*trans*-retinal (*B*), all-*trans*-retinol (*C*), and all-*trans*-retinyl ester (*D*) were quantified using UPLC. Total retinoid levels (*E*) are also presented. The mice carried the Rpe65-450Methionine variant. Mean ± SD is based on 5 to 8 samples, one eye per sample; ∗*p* <0.01, unpaired two-tailed *t* test.

Serial retinoid levels were also compared in *Rbp4*^*−/−*^ mice (agouti, Rpe65-450Met) *versus* C57BL/6J-Aw-J/J mice at ages 2, 5, and 8 months (Fig. 2). At age 2 months 11-*cis*-retinal was reduced by 77% (*p* < 0.01, unpaired two-tailed *t* test) in the *Rbp4*^*−/−*^ mice but by 5 months of age, approached WT levels in C57BL/6J-Aw-J/J mice. The decline in 11-*cis*-retinal at age 8 months is likely attributable to photoreceptor cell degeneration observed by this age (presented below). All-*trans*-retinol was significantly lower in the *Rbp4*^*−/−*^ mice at all of the ages studied and all-*trans*-retinyl ester was profoundly reduced.

### ERG recordings

**Figure 3:**
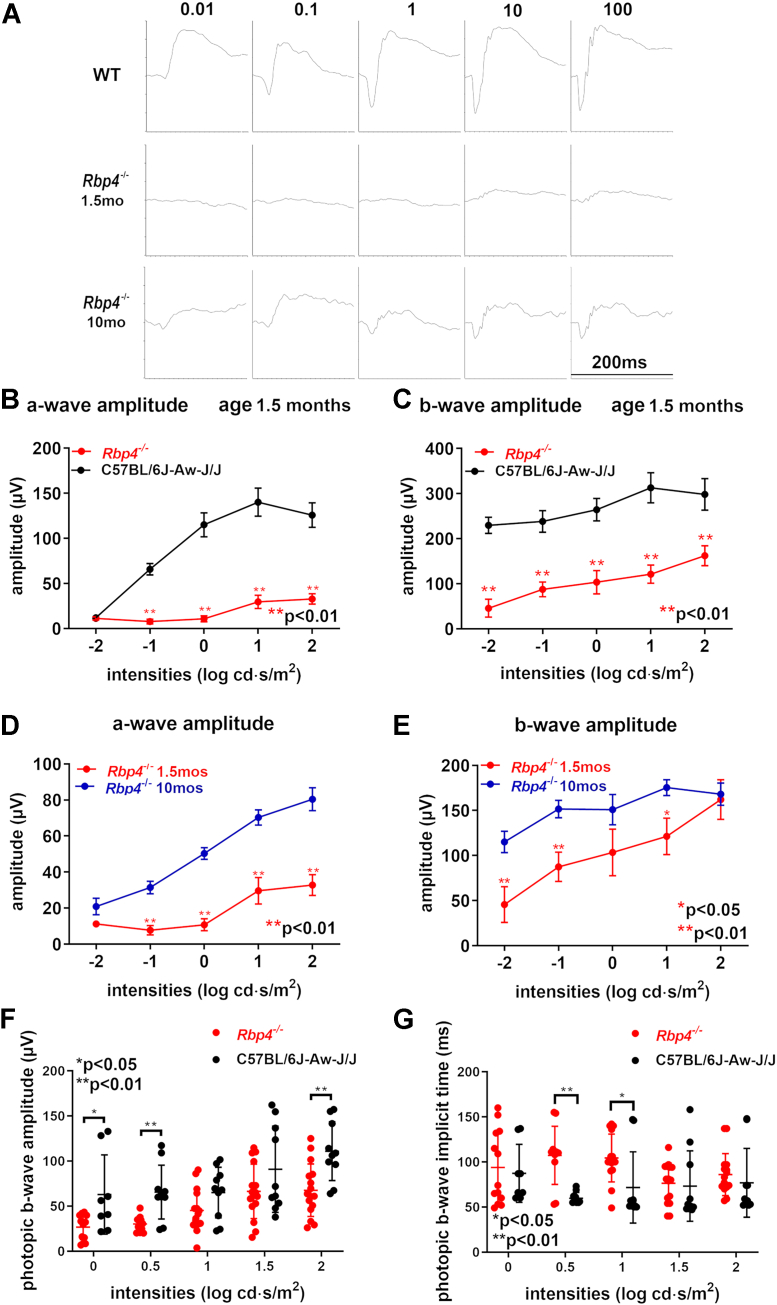
**Retinal function in *Rbp4***^***−/−***^**mice.***A*, representative scotopic electroretinograms (ERGs) recorded at light intensities of increasing strength (0.01–100 cd·s/m^2^). *B* and *C*, scotopic ERG waveforms recorded in 1.5-month-old *Rbp4*^*−/−*^ and WT (C57BL/6J-Aw-J/J) mice (Rpe65-450Met variant) and plotted as a function of single flash intensities. *D* and *E*, ERG a-wave and b-wave amplitudes presented for comparison of 1.5- and 10-month-old *Rbp4*^−/−^ mice. Mean ± SEM is based on 8 to 12 eyes. ∗*p* <0.05; ∗∗*p* <0.01, unpaired two-tailed *t* test. *F* and *G*, photopic b-wave amplitude and implicit times, age 2 months. Mean ± SEM, eight agouti *Rbp4*^*−/−*^ mice, and five agouti C57BL/6J-Aw-J/J mice are used.

To evaluate the function of photoreceptor cells (27) in *Rbp4*^*−/−*^ mice, scotopic ERG analysis was conducted at age 1.5 and 10 months. As shown in Figure 3, in agouti C57BL/6J-Aw-J/J WT mice, the amplitude of the a-wave increased with increasing light stimulus from threshold until saturation at 1.0 log cd⋅s/m^2^. The amplitudes of the a- and b-waves at age 1.5 months were appreciably lower (*p* < 0.01, unpaired two-tailed *t* test) in the *Rbp4*^*−/−*^ mice relative to the WT control mice (Fig. 3, *B* and *C*). Comparison of age 1.5 months *versus* 10 months old agouti *Rbp4*^*−/−*^ (Rpe65-450Met) mice revealed an improvement in a-wave and b-wave amplitudes by age 10 months (*p* < 0.01 for a-wave, *p* < 0.05 for b-wave, unpaired two-tailed *t* test) (Fig. 3, *D* and *E*). For instance, at a stimulus intensity of 1.0 log cd⋅s/m^2^, the increase in b-wave robustness between age 1.5 and 10 months was 46% (*p* < 0.05, unpaired two-tailed *t* test) (Fig. 3*E*). The significantly lower rod-dominated a-wave and b-wave amplitudes were consistent with the reduction in 11-*cis*-retinal (Fig. 1). A comparison of scotopic *versus* photopic responses suggested that the effect of Rbp4 deficiency had a greater impact on rods than cones (Fig. 3, *C* and *F*). Specifically, at stimulus intensities of 1.0 and 2.0 log cd⋅s/m^2^, the *Rbp4*^*−/−*^ mice presented with percent decreases in photopic b-wave amplitudes of 31% and 39%, while the scotopic b-wave amplitudes were 61% and 46% lower in the *Rbp4*^−/−^ mice.

### Assessment of photoreceptor cell viability

**Figure 4:**
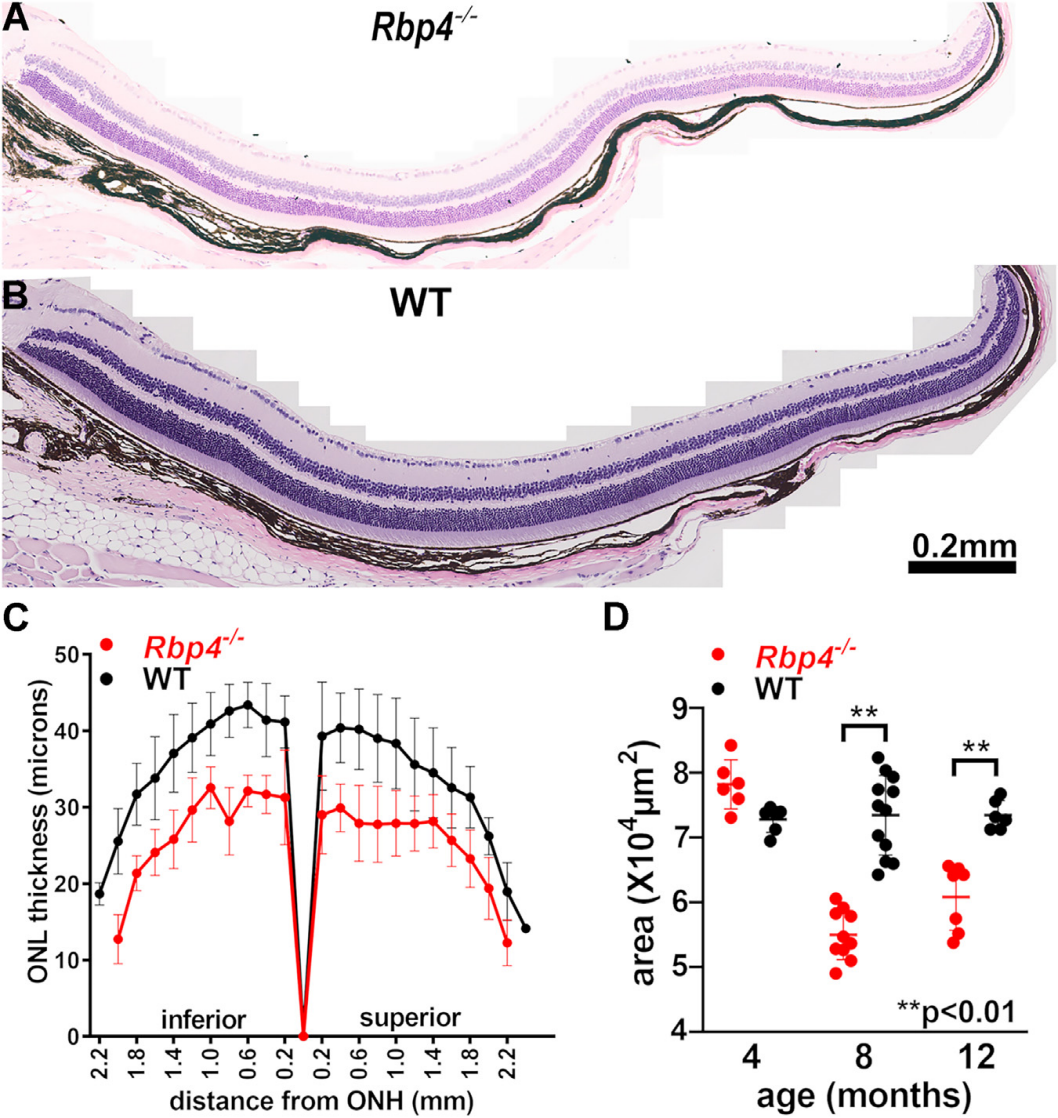
**Photoreceptor loss in retinas of adult *Rbp4***^***−/−***^**mice.***A* and *B*, representative H&E–stained sections of the retinas of 8-month-old *Rbp4*^*−/−*^ and WT (C57BL/6J-Aw-J/J) mice, inferior quadrant. Optic nerve head (ONH) is on the *left*. Mice had an agouti coat and carried the Rpe65-450Met variant. *C*, plots of outer nuclear layer (ONL) thickness as a function of distance from the ONH. *D*, ONL areas calculated from thicknesses 2-mm superior and inferior to the ONH. Mean ± SD, 6 to 12 eyes. ∗∗*p* < 0.01, unpaired two-tailed *t* test.

To evaluate photoreceptor cell viability, we measured outer nuclear layer (ONL) thicknesses (Fig. 4, *A* and *B*). To acquire values for statistical analysis, we calculated ONL area as the sum of thickness measurements from 0.2 to 1.0 mm from optic nerve head (ONH) multiplied by the interval of 0.2 to 2.0 mm (Fig. 4, *A* and *B*). ONL area in agouti *Rbp4*^*−/−*^ mice (Rpe65-450Met) was not reduced in thickness at 4 months of age but at 8 and 12 months of age, ONL was significantly thinner than controls (*p* < 0.01, unpaired two-tailed *t* test) (Fig. 4*C*). This finding is indicative of photoreceptor degeneration in the mutant mice.

### Fundus autofluorescence imaging

**Figure 5:**
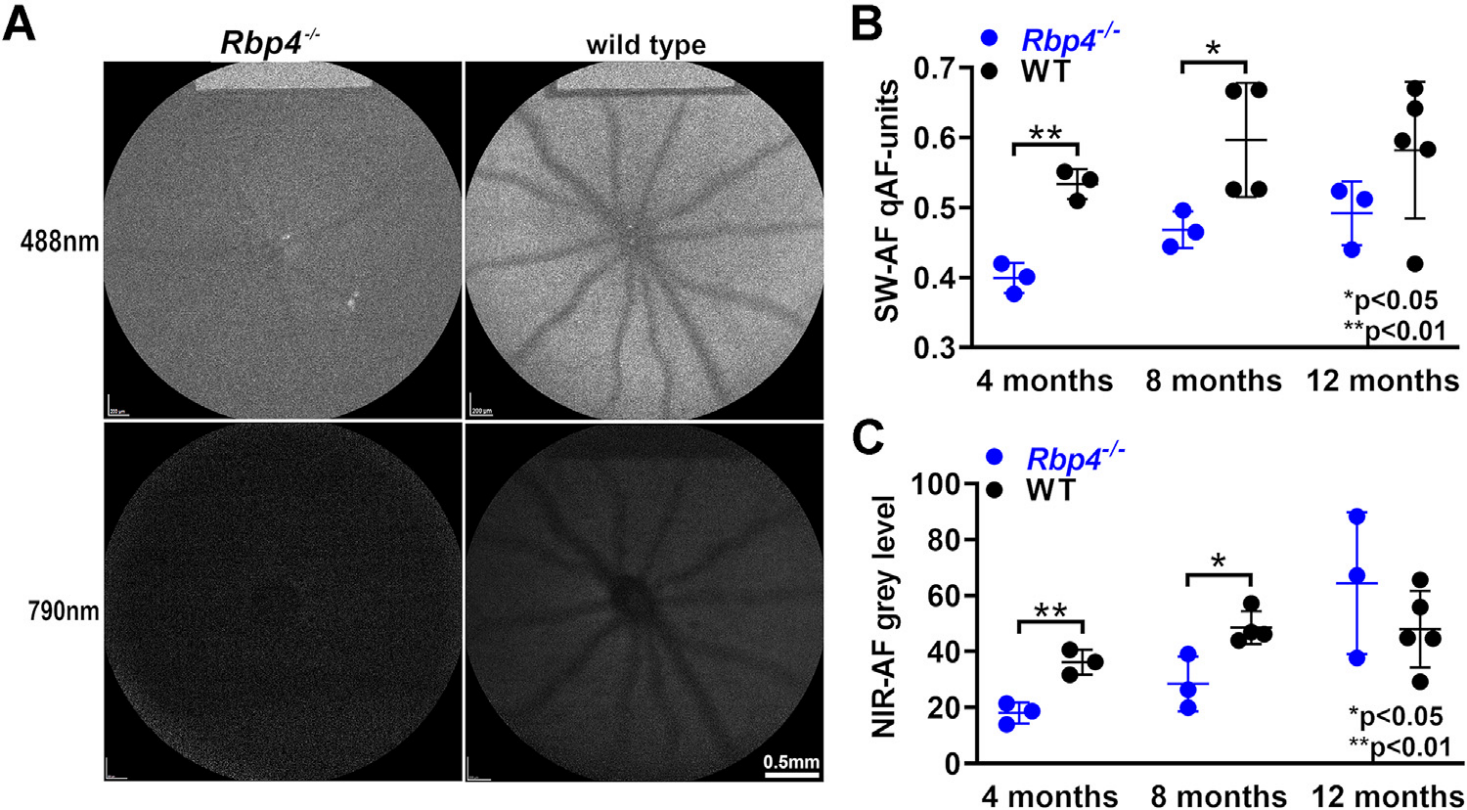
**Fundus autofluorescence imaging of *Rbp4***^***−/−***^**mice.***A*, fundus images acquired from age 4 months agouti *Rbp4*^*−/−*^ and WT (C57BL/6J-Aw-J/J) mice (Rpe65-450Met variant) at 488-nm (short-wavelength autofluorescence, SW-AF) and 790-nm (near infrared autofluorescence, NIR-AF) excitation. *B*, SW-AF intensities calculated as quantitative fundus autofluorescence (qAF) at indicated ages. Mean ± SD based on 6 to 10 eyes. *C*, measurements of NIR-AF in *Rbp4*^*−/−*^ and WT mice at indicated ages. ∗*p* < 0.05 and ∗∗*p* < 0.01 as compared to WT mice; 1-way ANOVA and Tukey’s multiple comparison test.

As a consequence of random nonenzymatic reactions of vitamin A aldehyde, bisretinoid fluorophores accumulate as the lipofuscin of retina (11). Fundus autofluorescence acquired with 488-nm excitation originates primarily in RPE bisretinoid lipofuscin. Using an established approach (23), we measured fundus AF levels noninvasively as quantitative fundus autofluorescence (qAF) in agouti *Rbp4*^*−/−*^ (Rpe65-450Met) mice ages 4, 8, and 12 months (Fig. 5). The experiments revealed that SW-AF was recordable in the *Rbp4*^*−/−*^ mouse; this finding is indicative of a functioning visual cycle. However, qAF was significantly lower in *Rbp4*^*−/−*^ mice *versus* WT C57BL/6J-Aw-J/J mice at ages 4 and 8 months (Fig. 5*B*). Nevertheless, there was a significant increase (17%) in qAF in *Rbp4*^*−/−*^ mice from age 4 months to 8 months (*p* < 0.05, two-tailed *t* test) and qAF increased by 23.2% between 4 and 12 months of age (Fig. 5*B*). Near infrared autofluorescence (NIR-AF) intensities paralleled the lower qAF values observed in *Rbp4*^*−/−*^ mice relative to the WT control (Fig. 5*C*).

### HPLC quantitation of bisretinoid

**Figure 6:**
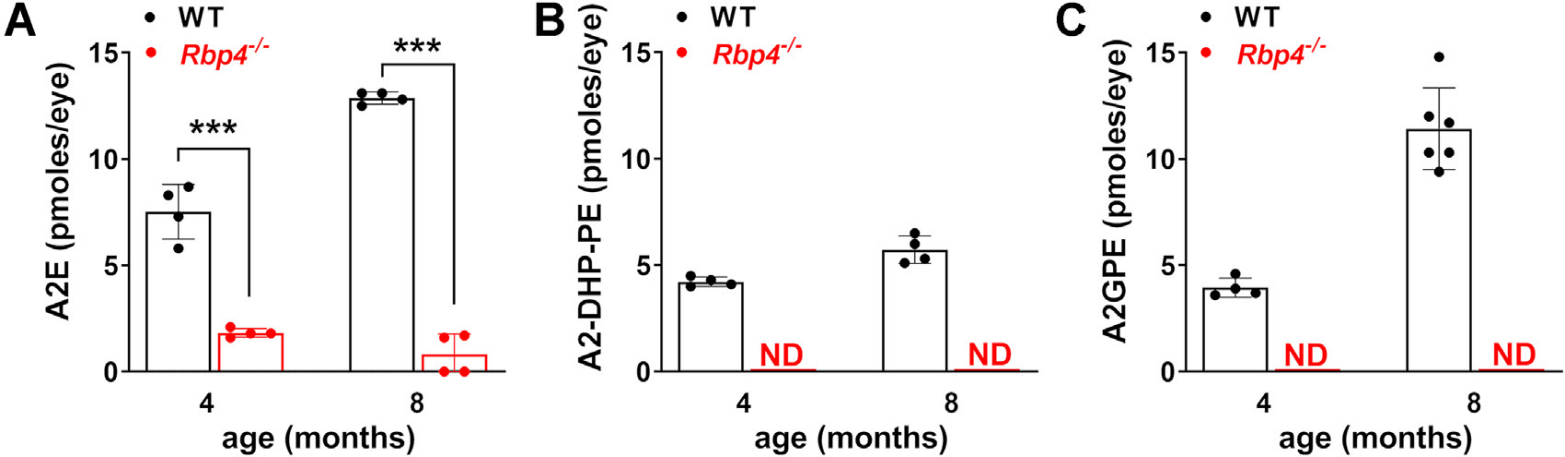
**HPLC quantification of bisretinoids.** A2E (*A*) A2-DHP-PE (*B*) and A2GPE (*C*). *Rbp4*^*−/−*^ and WT (C57BL/6J-Aw-J/J) mice were measured at ages 4 months and 8 months. Mice carried the Rpe65-450Met variant. Mice had an agouti coat-color, except for A2GPE analysis *Rbp4*^*−/−*^ mice had a black coat. Values are mean ± SD, 3 to 4 eyes/sample; n = 4. ∗∗∗*p* <0.001, 2-way ANOVA and Sidak’s multiple comparison test. ND, not detected. PE, phosphatidylethanolamine.

In eyes obtained from *Rbp4*^*−/−*^ mice (Rpe65–450Met; agouti), we measured bisretinoids by integrating HPLC peak areas and normalizing to standard samples of known concentration. At 4 months of age, the bisretinoid A2E was reduced by 76% (*p* < 0.001, 2-way ANOVA and Sidak’s multiple comparison test) in *Rbp4*^*−/−*^ relative to C57BL/6J-Aw-J/J WT control mice (Fig. 6*A*). At this age, the bisretinoid A2-DHP-PE levels were 4.2 pmoles/eye in the WT and nondetectable in the *Rbp4*^*−/−*^ mouse (Fig. 6*B*). In *Rbp4*^*−/−*^ mice at 8 months of age, A2E was reduced by 87% (*p* < 0.001, 2-way ANOVA and Sidak’s multiple comparison test) relative to WT control mice (Fig. 6*A*). Again, the bisretinoids A2-DHP-PE and A2GPE were not detected at 8 months of age in the *Rbp4*^*−/−*^ mouse, while the WT levels were 5.7 pmoles/eye and 11.4 pmoles/eye, respectively (Fig. 6, *B* and *C*).

### Adipose tissue–specific human RBP4 knock-in

While Rbp4 is predominantly produced by hepatocytes (28), Rbp4 can also be synthesized by adipocytes (1). Thus, we also studied transgenic mice that express human RBP4 (hRBP4) specifically in adipocytes (*hRBP4*^*+/+*^*AdiCre*^*+/−*^) (29). When fed a standard chow diet, these mice exhibit an elevation in adipose-derived total Rbp4 protein (mRbp4+ hRBP4) without significant differences in plasma Rbp4 or retinoid levels (retinol, retinyl ester, and all-*trans*-retinoic acids) nor differences in hepatic or adipose retinoid (29). Since plasma RBP4 levels are known to be increased in *hRBP4*^*+/+*^*AdiCre*^*+/−*^ mice fed a high fat diet, we also employed the latter diet providing 60% of calories as fat. Nevertheless, we did not observe elevations in ocular retinoids (11*cis*RAL, atROL, atRAL, and atRE) in *hRBP4*^*+/+*^*AdiCre*^*+/−*^ as compared to control mice (*hRBP4*^*+/+*^*AdiCre*^*−/−*^, *hRBP4*^*+/−*^*AdiCre*^*+/−*^) in mice fed a high fat diet (*p* > 0.05, 2-way ANOVA and Sidak’s multiple comparison test) (Fig. 1*D*).

### Developmental defects in *Rbp4*^*−/−*^ mice

**Figure 7:**
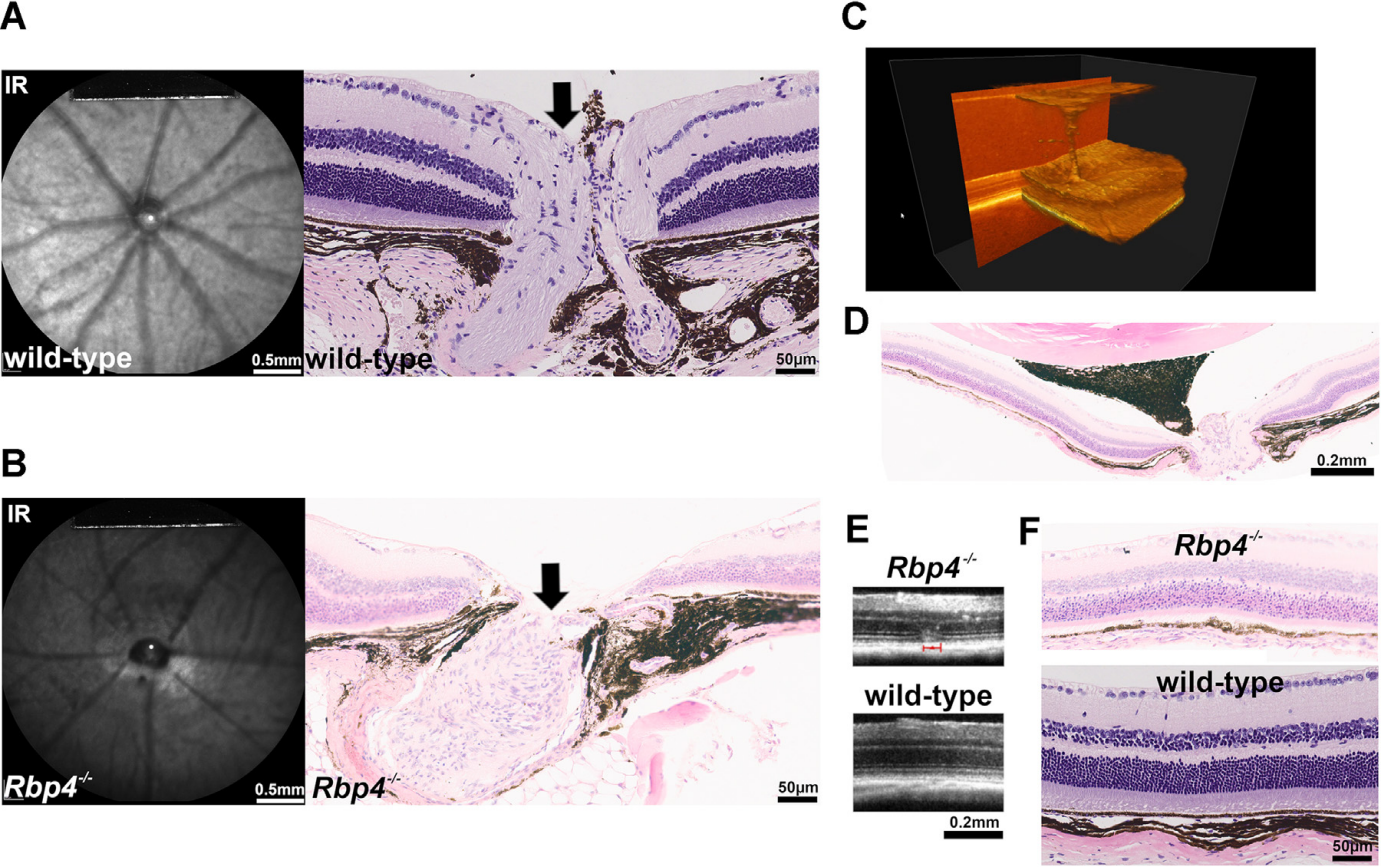
**Developmental defects in posterior segments of *Rbp4***^***−/−***^**mice.** Ages 4 months (*C* and *E*), 8 months (*A* and *B*), and 12 months (*D* and *F*). *A* and *B*, representative fundus infrared reflectance images (*left*) and photomicrographic images of retina (*right*) of WT (*A*) and *Rbp4*^*−/−*^ (*B*) mice. Optic nerve head (*black arrow*). *D*, persistent hyaloid artery extending to the back of the lens capsule as shown in a 3-D OCT image (*C*) and in a histological section (*D*). *E*, SD-OCT scans of *Rbp4*^*−/−*^ and WT mice with inferior retina. In the SD-OCT of an *Rbp4*^*−/−*^ mouse, a hyper-reflective lesion extends through photoreceptor cell-attributable layers and into the ONL (*red bar*). *F*, histological sections of retina acquired from *Rbp4*^*−/−*^ and WT mice (superior retina). Multiple layers of hypopigmented retinal pigment epithelial cells (RPE) and thinning or absence of choroidal layers in *Rbp4*^*−/−*^ mice. Mice carried the Rpe65-450Met variant. All WT mice were C57BL/6J-Aw-J/J. ONL, outer nuclear layer; SD-OCT, spectral domain optical coherence tomography.

The *Rbp4*^*−/−*^ mouse colony exhibited impaired breeding with an average of two or three surviving pups per litter as compared to 4 to 8 pups in control litters. About 40% of the mice had small lids openings, optic disc abnormalities and persistent hyaloid arteries often occurred (Fig. 7, *A* and *B*). On occasion, a hyaloid artery pulsing with blood was found by *in vivo* Doppler spectral domain optical coherence tomography. Histological analysis confirmed a hyaloid artery remnant extending from the optic disc to the back of the lens capsule (Fig. 7, *C* and *D*). In some cases, the ONH appeared abnormally concave and ectopic pigmentation was a feature. Multiple layers of hypopigmented retinal pigment cells were also observed in histological sections of the eyes (Fig. 7*F*). As noted earlier, a developmental deficiency in photoreceptor cell numbers was not detected at 4 months of age (Fig. 4, *A* and *B*) but photoreceptor cell loss was measured at age 8 months.

## Discussion

The *Rbp4*^−/−^ mouse is phenotypically normal except for the eye (6). Several years ago, investigators reported that mice deficient in retinol-binding protein (subsequently referred to as RBP4) presented with markedly reduced circulating retinol levels and impaired visual function measured as ERG b-wave amplitudes (9). The latter recordings revealed that the response to maximal stimulus was half of normal at age 1 month. At that age, ocular retinol levels were approximately 30% of WT but retinal histology was normal. However, when supplied with a vitamin A sufficient diet, b-wave amplitudes in the mutant mice progressively increased and reached amplitudes equal to that of WT mice by approximately 3 months of age. The ocular phenotype of *Rbp4*^−/−^ mice described by others (30) has been similar although b-wave amplitudes in the *Rbp4*^−/−^ mice remained at 50% of WT at age 10 months. Measurements of plasma retinol remained low in both studies while retinol levels in liver were higher in the *Rbp4*^*−/−*^ mice. The latter increase is explained by the failure of retinol to be released into the circulation due to the absence of Rbp4.

Here, we present new findings that further extend our understanding of vitamin A delivery to the retina. We observed that ocular 11-*cis*-retinal at age 4 months was reduced by 43% under conditions of Rbp4 deficiency; all-*trans*-retinyl ester, the storage form of retinoid in RPE, was 82% lower than WT. The lower levels of 11-*cis*-retinal in the mutant mice were accompanied by reduced rod-dominated a-wave and b-wave amplitudes at 1.5 and 10 months of age. Moreover, the effect on rod function appeared to be greater than the effect on photopic cone responses. This may be explained by the fact that while the classical visual cycle operates in the RPE to supply 11-*cis*-retinal to both rods and cones, an additional visual cycle exists in Müller glial cells for the purpose of delivering additional 11-*cis* chromophore to cones (31). Since there was no evidence of photoreceptor cell degeneration at 4 months of age, the reduction in scotopic ERG amplitudes was attributable to reduced 11-*cis*-retinal rather than photoreceptor cell degeneration. Nevertheless, at ages 8 and 12 months, photoreceptor cell death was evidenced by ONL thinning and by a downturn in 11-*cis*-retinal. These findings are consistent with the progressive retinal degeneration observed in human subjects in association with homozygous splice site mutations in RBP4 (32, 33).

In the setting of a polymorphism in the visual cycle isomerase Rpe65, replacement of leucine by methionine at amino acid 450 retards the regeneration of 11-*cis*-retinal probably by reducing Rpe65 protein stability (24, 25). Consequently, we have observed that 11-*cis*-retinal levels are appreciably lower (55%) in cyclic light-adapted albino mice expressing the Rpe65-450Met variant as opposed to leucine (26). Interestingly, however, in the *Rbp4*^*−/−*^ mice, the difference in 11-*cis*-retinal levels between Rpe65-Leu450 mice *versus* Rpe65-450Met mice was only 8% due to the impairment caused by the Rbp4 deficiency. Since on the *Rbp4*^−/−^ background, the visual cycle is not functioning at full capacity, the difference in 11-*cis*-retinal levels due to the variant was not realized.

We observed a significant decrease in the bisretinoids A2E, A2GPE, and A2-DHP-PE in *Rbp4*^*−/−*^ as compared to wild-type mice at both 4 and 8 months of age with quantitation by HPLC. An age-related increase from 4 to 8 months in the *Rbp4*^*−/−*^ mice was not observed. The decrease is consistent with noninvasive SW-AF measurements performed in this study. The decrease in A2E, A2GPE, and A2-DHP-PE in the setting of Rbp4 absence is not surprising, since it is known that bisretinoid formation is modulated by changes in the kinetics of the visual cycle (17, 18, 19, 21, 34). The NIR-AF elicited at the fundus originates primarily from RPE melanin (35). The reduction in NIR-AF observed in *Rbp4*^*−/−*^ mice is consistent with our observation that bisretinoids modulate the NIR-AF signal (36). It has been estimated that 10 to 20% of the body’s vitamin A is stored as retinyl esters in adipocytes (37). In transgenic mice that express human RBP4 (hRPB4) in adipocytes, total RBP4 (mRbp4 and hRBP4) protein is increased in adipose tissue but retinol is not increased in liver nor visceral adipose tissue (29). Fasting plasma free fatty acids are elevated in chow-fed adi-hRBP4 mice, and elevated hepatic triglyceride levels are considered to result from increased hepatic uptake of adipose-derived circulating free fatty acids (due to hydrolysis of adipose triglyceride) that enable increased free fatty acid availability for hepatic triglyceride synthesis. Neither plasma RBP4 nor retinol are observed in these mice. This may explain why we found that high levels of total RBP4 production in adipocytes did not confer elevated retinoids in retina.

Rbp4 deficiency was not associated with reduced ONL thickness in the young mice (age 2 months); this finding indicates that photoreceptor cell development is not limited by the availability of Rbp4. However, other developmental abnormalities included irregularities in the RPE monolayer and the ONH along with persistent hyaloid arteries all of which have been previously reported (30). It will be interesting to learn how Rbp4 interacts with other signaling molecules to enable formation of the optic nerve (38). In humans, missense mutations in *RBP4* are associated with ocular malformations of varying severity due to insufficient retinoic acid synthesis from vitamin A. For instance, mutant proteins carrying threonine substitutions at positions 55 and 57 (A55T, A57T) are known to form complexes with TTR and have increased affinity for the cell surface receptor STRA6 but they carry little vitamin A due to impaired binding (39). These dominant negative RBP4 proteins impact vitamin A delivery in both the fetus and at the placenta with the result that inheritance from the mother is greater than from the father. This mode of recessive inheritance of Rbp4 impairment has also been studied in a canine model (40).

In summary, we find that knock-out of Rbp4 is associated with appreciable reductions in 11-*cis*-retinal and all-*trans*-retinyl ester and associated impairment in visual function. Interestingly, however, despite the absence of Rbp4, the only known carrier of all-*trans*-retinol in the circulatory system (5), ocular retinoid levels and ERG amplitudes improve with age as the retina obtains vitamin A by non-Rbp4 mechanisms. Nevertheless, histological analyses revealed that the deficiency in retinoid ultimately leads to the degeneration of photoreceptor cells by age 8 months in the *Rbp4*^*−/−*^ mice. We have shown previously that qAF can reflect vitamin A deficiency after bariatric surgery (41) and provides feedback on visual cycle activity after RPE65 gene therapy (42). Similarly, recording of the SW-AF signal in the *Rbp4*^*−/−*^ mouse was also indicative of a functioning visual cycle. Bisretinoid fluorophores that give origin to SW-AF were diminished in amount in part because the measurement of these fluorophores included early ages when retinoid were particularly low. These issues are relevant to efforts being made to develop therapeutics that target the visual cycle (43).

## Experimental procedures

### Animals

Agouti *Rbp4*^*−/−*^ mice were bred in-house. These mice carried either the Rpe65-Leu450, the Rpe65-450Met variant or were heterozygous (Rpe65-Leu450Met). Control mice were either *Rpb4*^*+/+*^ litter mates or C57BL/6J-Aw-J/J mice (Rpe65-450Met; agouti) purchased from The Jackson Laboratory as controls. We also studied mice that express human *Rbp4* (hRBP4^+/+^AdiCre^+/+^) and the control mice (hRBP4^+/+^AdiCre^−/−^, hRBP4^−/−^AdiCre^+/+^) (29). Mice were fed a standard chow diet that included vitamin A (15 IU/gm). *Rbp4*^*−/−*^ breeders were fed LabDiet #5058 (vitamin A 15 IU/gm) or with a supplementation of vitamin A to bring its level to 30 IU/gm. Mice expressing hRBP4 (hRBP4^+/+^AdiCre^+/+^) and the control mice (hRBP4^+/+^AdiCre^−/−^, hRBP4^−/−^AdiCre^+/+^) were fed a high fat diet D12492 (60 kcal% fat and 4 IU/gm vitamin A) for 10 weeks. Mice were free of the Crb1/Rd8, rd1, rd2, rd3, rd6, rd7, and rd10 mutations. Animal protocols were approved by the Institutional Animal Care and Use Committee of Columbia University and complied with guidelines set forth by the ARVO Animal Statement for the Use of Animals in Ophthalmic and Vision Research.

### Fundus imaging

SW-AF images were captured using 488-nm excitation in anaesthetized mice (23, 26) using a confocal scanning laser ophthalmoscope (Spectralis HRA, Heidelberg Engineering). SW-AF intensities (qAF) were calculated from mean gray levels with normalization to an internal reference. NIR-AF (790-nm excitation) images were recorded as a mean of 100 frames obtained using the high-resolution automatic real-time mode of the Spectralis. Doppler recordings were acquired by SD-OCT (Bioptigen, Leica Microsystems) to assess the optical nerve head and hyaloid vessels in *Rbp4*^*−/−*^ mice (Rpe65-450Met, agouti).

### Electroretinography

Full field ERGs were recorded in dark-adapted (18 h) mice (Rpe65-450Met, agouti) at the indicated ages. The mice were anaesthetized with an intraperitoneal injection of a mixture of ketamine (80 mg/kg), xylazine (10 mg/kg), and acepromazine (5 mg/kg). Pupils were dilated with topical phenylephrine hydrochloride and tropicamide and body temperature was maintained at 35 to 37 °C. Recordings were acquired with a Celeris rodent ERG system (Diagnosys) and a pair of corneal electrodes positioned with GenTeal Tears gel (Alcon Laboratories). Scotopic ERG responses in dark-adapted mice were elicited by single flashes having intensities from 0.01 cd·s/m^2^ to 100 cd·s/m^2^. Photopic responses from 0.1 to 100 cd⋅s/m^2^ intensities were recorded in mice adapted to background light of 30 cd⋅s/m^2^ (10 min). Triplicate responses were computer-averaged for each flash condition. The amplitude of the a-wave was measured from the baseline to the first trough. The amplitude of the b-wave was measured from the trough of the a-wave to the following positive peak.

### Histology

Sagittal 5 micron H&E-stained paraffin sections most centrally located within the ONH were imaged digitally. ONL thickness was measured at 200-micron intervals in the vertical plane and plotted as distance (mm) superior and inferior to the ONH (44). ONL area was calculated as the measurement interval of 0.2 mm multiplied by the sum of ONL thicknesses in superior and inferior hemiretina. High resolution images were examined for histological change in and around the retina.

### Quantitative HPLC and UPLC

For analysis of retinoids (45), mouse eyes (1 eye/sample) frozen in liquid nitrogen were homogenized in PBS containing 100 mM *O*-ethylhydroxylamine·HCl, neutralized to a pH 6.5 with 4N NaOH. After the addition of 1 ml methanol, all-*trans*-retinol acetate was added as an internal standard. Hexane was added and the sample was vortexed in order to extract retinoids. After solubilization in hexane and centrifugation, the sample was dried under argon gas and redissolved in acetonitrile. The sample was injected into a reverse phase column (CSH C18 column, Waters) for elution in a Waters Acquity UPLC system using gradients of water (A) and acetonitrile (B) containing 0.1% of formic acid as follows: 0 to 5 min, 60% B; 5 to 60 min, 60 to 70% B; 60 to 70 min, 70 to 100% B; and 70 to 90 min, 100% B min (flow rate of 0.3 ml/min). Retinal (*O*-ethyl) oximes (11-*cis*-retinal and all-*trans*-retinal) were monitored at 360 nm, and all-*trans*-retinol and all-*trans*-retinyl palmitate were monitored at 320 nm. UV absorbance peaks were identified by comparison with external standards of synthesized retinoids.

For quantitation of bisretinoids, mouse eyes (4 eyes/sample) were homogenized and extracted in chloroform/methanol (1:1) and after filtering, the solvent was evaporated as previously described (46). The extract was redissolved in chloroform/methanol and bisretinoids were measured by HPLC (Waters Corp) (19). The samples were injected into a reverse phase column (Atlantis dC18, Waters) for elution in a Waters Alliance HPLC system using a gradient of acetonitrile in water with 0.1% TFA: 75 to 90% acetonitrile (0–30 min); 90 to 100% acetonitrile (30–40 min); 100% acetonitrile (40–80 min) with a flow rate of 0.5 ml/min for the mobile phase. Absorbance peaks were identified by comparison with external standards and molar quantities per eye were calculated by comparison to standard concentrations determined spectrophotometrically using published extinction coefficients and normalization to total sample volumes. Four eyes were combined for bisretinoids measurement, each measurement was expressed as picomoles/eye, and from multiple measurements, mean values were determined.

### Statistical analysis

Statistical analysis was performed using GraphPad Prism version 9 (GraphPad Software, Inc); *p* < 0.05 was considered significant.

## Data availability

All of the data are in the manuscript.

## Conflicts of interest

The authors declare that there are no conflicts of interest related to the contents of this article. The content is solely the responsibility of the authors and does not necessarily represent the official views of the National Institutes of Health.
